# Pharmacological and Mechanistic Interventions for Cognitive Impairment Associated With Schizophrenia: A Review of Registered Clinical Trials

**DOI:** 10.1111/acps.70034

**Published:** 2025-09-04

**Authors:** Bahareh Peyrovian, Lena Palaniyappan, Theodore T. Kolivakis, Howard C. Margolese

**Affiliations:** ^1^ Department of Psychiatry McGill University Health Centre, Allan Memorial Institute Quebec Canada; ^2^ Department of Psychiatry Douglas Research Centre, McGill University Quebec Canada

**Keywords:** clinical trials, cognition, neuromodulation, psychopharmacology, schizophrenia, treatment

## Abstract

**Background:**

Schizophrenia is characterized by positive, negative, and cognitive symptoms. Current pharmacological treatments often fail to address cognitive deficits. In this review of clinical trials, we aim to identify studies that explore neurobiological (non‐psychological) strategies to address Cognitive Impairment Associated with Schizophrenia (CIAS).

**Methods:**

A search of clinical trial databases was conducted through US National Institutes of Health's ClinicalTrials.gov and the World Health Organization's International Clinical Trials Registry Platform (ICTRP) on August 2, 2024, with complementary searches performed on July 4 and 10, 2025, for each respective database to update the results.

**Results:**

We identified 510 relevant interventional studies that objectively measured cognitive performance. Most trials were conducted in the United States (36.4%) and focused on treatment (79%), with randomized designs (88%), investigating drugs (56%), devices (33%), and dietary supplements (10%). Of these trials, 17% reported positive pro‐cognitive evidence. Glutamate modulators were the most studied drug category (63 trials), with positive results for sarcosine, BI425809 (Iclepertin), d‐serine, d‐cycloserine, and minocycline in small‐scale trials, although the results were not replicated in larger studies. Nicotinic receptor modulators like ABT‐126 and encenicline also showed some cognitive benefits. Device‐based interventions, particularly rTMS and iTBS, demonstrated improvements in global cognition, working memory, attention, and processing speed in a subset of trials.

**Conclusion:**

In this comprehensive overview of clinical trials on pro‐cognitive agents in schizophrenia, we identify emerging opportunities but also acknowledge a lack of replicated evidence. Despite extensive attempts to address CIAS, it remains an undertreated domain, and future trials should explore better ways to treat this important condition.

**Abbreviations**
5‐HT: 5‐hydroxytryptamine
BACS: brief assessment of cognition in schizophrenia
CANTAB: Cambridge neuropsychological test automated battery
CIAS: cognitive impairment associated with schizophrenia
DAAO: d‐amino acid oxidase
DBRCT: double‐Blind randomized clinical trials
DLPFC: Dorsolateral prefrontal cortex
FDA: food and drug administration
GABA: Gamma‐aminobutyric acid
GlyT1: Glycine transporter type 1
GMLT: Groton maze learning task
ICTRP: World Health Organization's International Clinical Trials Registry Platform
iTBS: intermittent theta burst stimulation
MATRICS: measurement of treatment research to improve cognition in schizophrenia
MCCB: MATRICS consensus cognitive battery
MoCA: Montreal Cognitive Assessment
NAC: *N*‐acetylcysteine
nAChRs: nicotinic acetylcholine receptors
NIMH: National Institute of Mental Health
NMDA receptors: *N*‐methyl‐d‐aspartate receptors
OCLT: one card learning task
PANSS: positive and negative syndrome scale
RBANS: repeatable battery for the assessment of neuropsychological status
rTMS: transcranial magnetic stimulation
rTUS: repetitive transcranial ultrasound stimulation
SCoRS: schizophrenia cognition rating scale
tACS: transcranial alternating current stimulation
tDCS: transcranial direct current stimulation
TMT: trail making test
WCST: Wisconsin card sorting test

**Summary**

- Significant outcomes
  ○ Various compounds with diverse mechanisms of action were investigated in CIAS, and some such as glutamate and nicotinic modulators showed cognitive benefits in specific domains. However, larger trials failed to demonstrate benefits.
  ○ Neuromodulation techniques, including rTMS and tDCS, demonstrate improvement in CIAS in small‐scale studies, which require replication in larger trials.
  ○ Future therapeutic strategies may benefit from integrative approaches combining pharmacological agents, neuromodulation, and cognitive remediation tailored to individual cognitive needs.
- Limitations
  ○ Only trials listed on ClinicalTrials.gov and the WHO ICTRP were included, potentially missing relevant studies from other resources.
  ○ Trials which did not list cognitive batteries as part of their protocol’s initial descriptions but had cognitive assessments in their study process were not identified in the primary database, which may have resulted in the exclusion of some trials.
  ○ The heterogeneous outcome measures, trial durations, and small sample sizes limit comparisons across studies.

## 1 Introduction

Schizophrenia is characterized by positive (e.g., hallucinations and delusions) and negative symptoms (e.g., social withdrawal, diminished emotional expression, poverty of speech and lack of spontaneity) as well as symptoms of cognitive impairments such as deficits in attention, processing speed, executive functions, psychomotor speed, memory, and social cognition [1, 2, 3].

The concept of cognitive impairment in schizophrenia dates to 1890s, when Emil Kraepelin, a German psychiatrist, defined schizophrenia as “dementia praecox,”, describing it a “tangible affection of the brain, probably damage or destruction of cortical cells…”. Eugen Bleuler in 1915 stated that “at least the great majority of clinical pictures, which are not collected under the name of dementia praecox, rests on some toxic action or anatomical process, which arises independently of psychic influences…” [4, 5]. These remarks underscore the initial understanding of distinct pathophysiology of cognitive impairment and psychotic symptoms in pathophysiology of schizophrenia.

Cognitive deficits in schizophrenia typically range from moderate to severe, with all individuals showing some impairment compared to their expected cognitive level [6]. These impairments often manifest early in the course of illness and sometimes precede the formal diagnosis of schizophrenia [7]. Despite treatment with antipsychotics, cognitive impairment frequently persists and imposes significant effects on functional outcomes and remains a barrier to recovery [8]. In the past two decades, with advancements in knowledge of the underlying pathophysiology, cognitive impairment associated with schizophrenia (CIAS) has gained recognition as a treatment target [9]. For instance, in order to standardize the assessment of CIAS, the MATRICS consensus cognitive battery (MCCB) was developed through a collaboration involving the National Institute of Mental Health (NIMH), FDA, academia, and industry [10]. In addition, CIAS has been endorsed by the Division of Psychiatry Products of the FDA as an indication for treatment development [11]. Despite these advances, no pharmacological compounds or other modalities besides psychological interventions have yet been approved for the treatment of CIAS. The barriers to developing effective treatment include, but are not limited to, the complex etiology [12] and heterogeneity both between and within individuals across cognitive domains and over time, ranging from global deficits to selective domain‐specific dysfunction [13, 14] as well as challenges in clinical trials—such as small sample sizes, short treatment durations, high placebo response rates [15, 16, 17], and lack of real‐world functional outcomes [18].

## 2 Aims of the Study

The primary goal of this review was to analyze clinical trials investigating novel, non‐psychological interventions for cognitive impairment associated with schizophrenia. We specifically focused on interventional trials using objective cognitive performance measures. The secondary goal was to evaluate the efficacy of these interventions and identify promising therapeutic directions.

## 3 Materials and Methods

### 3.1 Study Strategy

A search of the National Institutes of Health's ClinicalTrials.gov and the World Health Organization's International Clinical Trials Registry Platform (ICTRP) database was conducted on August 2, 2024, with additional complementary searches performed on July 4 and 10, 2025, for each respective database to update the results. The inclusion criteria were interventional clinical studies that examined novel non‐psychological interventions (defined as modalities not yet established in clinical practice for the treatment of schizophrenia) aimed at improving CIAS.

The studies objectively measured performance in at least one of the following MCCB domains: speed of processing, attention/vigilance, working memory, verbal learning, visual learning, reasoning and problem solving, or social cognition.

An original search of “cognitive impairment” and “schizophrenia” was conducted and cross‐referenced with the following terms: schizophrenia spectrum disorder and other psychotic disorders, schizophrenia disorder/s, psychosis, psychotic disorder/s and cognition, cognitive dysfunction, cognitive function, cognitive disability, cognitive deficits, cognitive enhancement, cognitive training, cognitive remediation, and cognitive rehabilitation among others.

### 3.2 Study Selection and Eligibility Criteria

**FIGURE 1:**
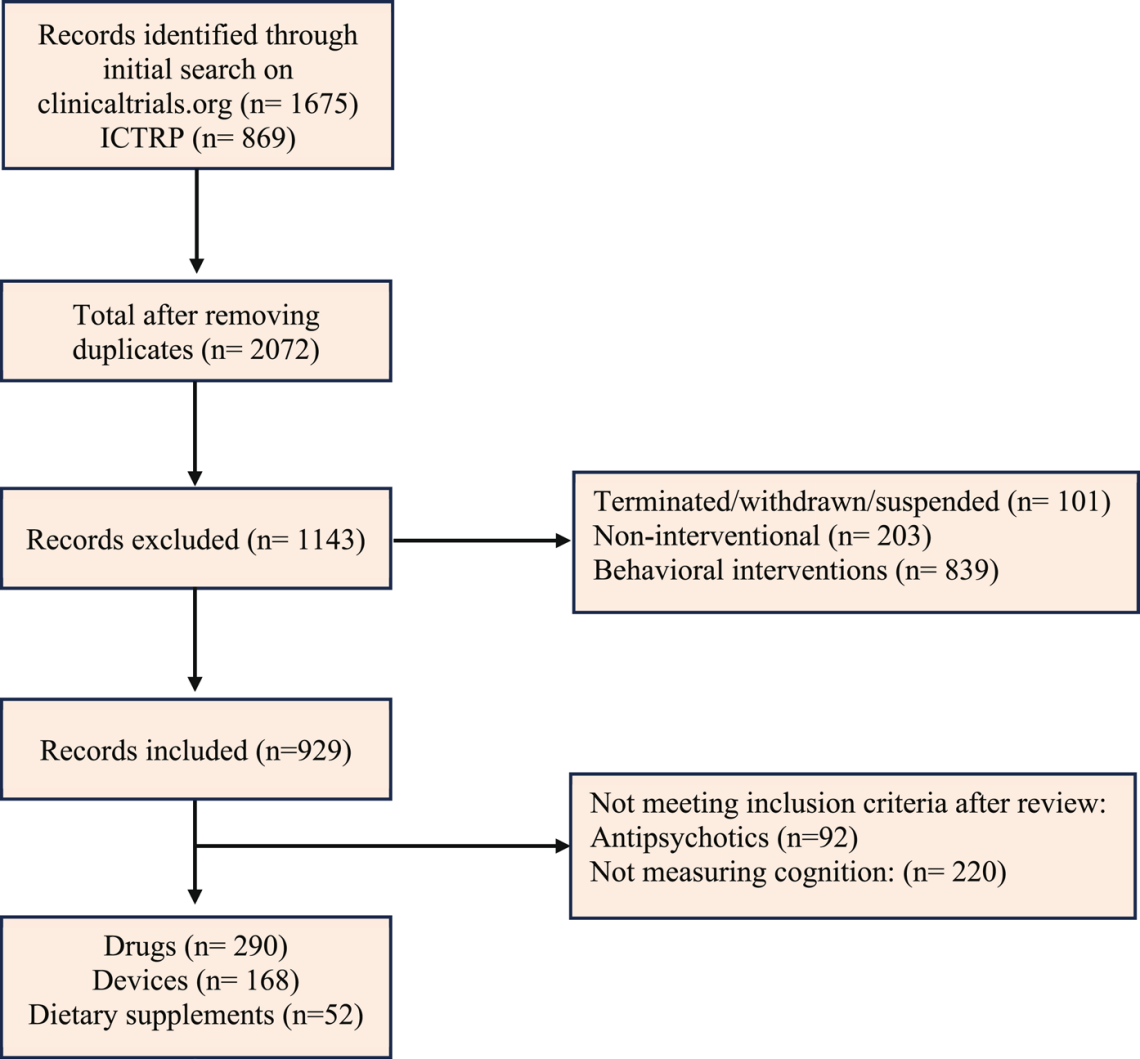
Study selection schema.

The study selection schema can be found in Figure 1. All completed, active, and upcoming clinical studies involving randomized and non‐randomized trials were identified and included in the current review. Mechanistic studies that recruited healthy participants alone, non‐experimental, basic science, and observational studies without interventions, behavioral intervention, and physical exercise as well as interventional studies that solely targeted negative symptoms but did not use a cognitive battery to estimate outcome measures were excluded. Likewise, studies using the Positive and Negative Syndrome Scale (PANSS) to report cognitive performance were excluded from the analysis, as PANSS does not measure MATRICS domains directly. Additionally, in order to identify novel treatment strategies, trials that investigated conventional antipsychotics were excluded from the analysis, even if they reported effects on cognition.

### 3.3 Data Extraction and Analysis

Data recorded for each clinical study included study type, phase, allocation, intervention model, masking, primary outcome, estimated start and end dates, actual completion duration, method of administration, dose, number of doses, drug type, control condition (if applicable), location, and sponsor. After the extraction of relevant clinical study parameters, data were summarized by using JASP statistical software (version 0.191.1) where needed. Trials with any positive pro‐cognitive results were chosen for further discussion. Research methods were adopted from studies with similar methods [19, 20, 21].

## 4 Results

Our search results returned 2072 clinical trials after removing duplicates. Non‐interventional studies, terminated, withdrawn, suspended studies, and behavioral interventions were excluded. The remaining clinical trials were manually reviewed to verify inclusion criteria, resulting in the exclusion of 92 trials investigating antipsychotics and 290 studies that did not include at least one domain of cognitive function in their trial descriptions. A total of 510 clinical trials were analyzed further for their characteristics.

### 4.1 Trials' Overall Characteristics

At the time of data extraction, one hundred eighty‐six (36.4%) studies were registered in the United States, 89 (17.4%) in China, and 31 (6.4%) involved multiple countries. The primary purpose of 405 (79.4%) was defined as treatment, 6 (1.1%) prevention, 20 (3.9%) basic science, 4 (0.7%) supportive care, 13 (2.5%) other, and 61 (11.9%) unknowns. Four hundred fifty (88.2%) were randomized trials, 13 (2.5%) non‐randomized, and the allocation was not defined in the study protocol in 47 (9.6%). Two hundred ninety (56.8%) studies investigated a drug, 58 (12%) a dietary supplement, and 144 (29.9%) trials studied various devices. In 460 (90.1%) trials, both male and female participants were included; in 8 (1.5%) only female, in 16 (3.1%) male, and in 22 (4.5%) the sex was not identified. In 292 (57.2%) studies, the participants were adults (18–65), in 165 (32.3%) adults and older adults (> 18), in 13 (2.5%) children and adults (< 65), in 1 (0.2%), only children (< 18), in 3 (0.5%) all age groups, and in 35 (6.7%) the age group was not defined.

**FIGURE 2:**
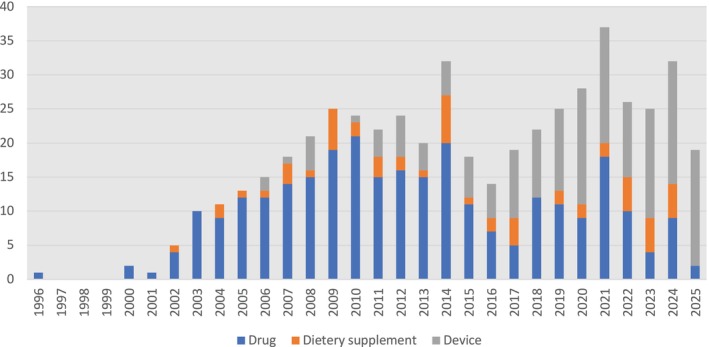
Number of new trials in each category of intervention.

Eight (1.6%) trials were indicated as phase 0, 8 (1.5%) were early phase 1, 26 (5%) phase 1, 123 (24.1%) phase 2, 15 (3%) phase 1/2, 47 (9.3%) phase 3, 13 (2.6%) phase 2/3, 56 (10.9%) phase 4, 3 (0.6%) phase 3/4 and 1(0.2%) were post‐marketing studies. At the time of data collection, only trials related to BI 425809 were in phase 3 for the indication of cognitive enhancer in schizophrenia, and the rest of the compounds in phases 3 and 4 were previously marketed for other indications. There has been an increase in the number of trials in the past 10 years, which is more prominent in the device category. Figure 2 provides an overview of the total number of trials registered in the database each year, categorized by intervention type. Ninety‐one (17.6%) of trials reported positive pro‐cognitive evidence in schizophrenia subjects. Table 1 provides details about the trials' overall characteristics in drug, device, and dietary supplement groups. The full list of trials with negative outcomes and trials without associated results is available in supplemental materials, Tables 1 and 2.

**TABLE 1:** Characteristics of studies.

Characteristics	Drug (*n* = 290)	Device (*n* = 168)	Dietary supplements (*n* = 52)	Total (*n* = 510)
Primary purpose				
Treatment	241 (83.1%)	122 (72.6%)	42 (80.7%)	405 (79.4%)
Prevention	3 (1%)	1 (0.5%)	2 (3.4%)	6 (1.1%)
Basic science	10 (3.5%)	10 (5.9%)	—	20 (3.9%)
Supportive care	1 (0.3%)	2 (1.1%)	1 (1.9%)	4 (0.8%)
Health‐service research	1 (0.3%)	—	—	1 (0.2%)
Other	8 (2.8%)	3 (1.7%)	2 (3.8%)	13 (2.6%)
Unknown	26 (8.6%)	30 (17.8%)	5 (9.6%)	61 (11.9%)
Allocation				
Randomized	265 (91.3%)	138 (82.1%)	47 (90.3%)	450 (88.2%)
Non‐randomized	6 (2.1%)	6 (3.5%)	1 (1.9%)	13 (2.5%)
Unknown	19 (6.85%)	24 (15.9%)	4 (6.8%)	47 (9.4%)
Interventional model				
Parallel	209 (72%)	126 (75%)	41 (78.8%)	376 (73.7%)
Crossover	50 (17.2%)	21 (12.5%)	5 (9.6%)	76 (14.9%)
Sequential	1 (0.3%)	3 (1.7%)	—	4 (0.8%)
Factorial	3 (1%)	1 (0.5%)	—	4 (0.8%)
Cross‐sectional	—	1 (0.6%)	—	1 (0.2%)
Single group	21 (7.3%)	15 (8.9%)	3 (5.7%)	39 (7.8%)
Dose comparison	1 (0.3%)	—	—	1 (0.2%)
Unknown	4 (1.7%)	1 (0.6%)	3 (5.7%)	9 (1.2%)
Masking model				
Quadruple	100 (35%)	20 (11.9%)	25 (48%)	145 (28.4%)
Triple	42 (14.4%)	20 (11.9%)	4 (7.6%)	66 (13%)
Double	98 (33%)	52 (30.9%)	12 (23%)	162 (31.7%)
Single	7 (2.4%)	20 (1.9%)	3 (5.7%)	30 (5.8%)
None (open label)	20 (6.8%)	15 (8.8%)	6 (11.5%)	41 (7.9%)
Unknown	23 (7.9%)	39 (20%)	2 (3.8%)	62 (12.2%)
Age group				
Adult (18–65)	164 (56.5%)	100 (59.5%)	28 (53.8%)	292 (57.7%)
Adult, older adult (> 18)	103 (35.5%)	43 (25.5%)	19 (36.5%)	165 (32.3%)
Older adults	—	1 (0.5)	—	1 (0.2%)
Child, adult (< 65)	6 (2.1%)	7 (4%)	—	13 (2.5%)
Children (< 18)	1 (0.3%)	—	—	1 (0.2%)
All age groups	—	1 (0.6%)	1 (1.9%)	3 (0.5%)
Unknown	15 (5.2%)	13 (8.9%)	4 (7.6%)	35 (6.8%)
Sex				
All	264 (91%)	148 (88.8%)	48 (92.3%)	460 (90%)
Female	7 (2.4%)	1 (0.6%)	—	8 (1.6%)
Male	11 (3.7%)	3 (2%)	2 (3.8%)	16 (3.1%)
Unknown	8 (2.8%)	16 (9.4%)	2 (3.8%)	26 (4.5%)
Phase				
0	—	7 (4.1%)	1 (1.9%)	8 (1.6%)
Early 1	8 (2.7%)	—	—	8 (1.4%)
1	22 (7.7%)	4 (2.3%)	—	26 (5%)
2	110 (37.8%)	5 (2.9%)	9 (17.3%)	124 (24%)
1/2	9 (3.1%)	2 (1.1%)	4 (7.6%)	15 (3%)
3	35 (12%)	4 (2.3%)	8 (15.3%)	47 (9. %)
2/3	7 (2.4%)	—	6 (11.5%)	13 (2.6%)
4	53 (18.5%)	1 (0.6%)	2 (3.8%)	56 (11.6%)
Other	1 (0.3%)	2 (1.3%)	—	3 (0.6%)
Post‐market	1 (0.3%)	—	—	1 (0.2%)
Unknown	42 (14.7%)	143 (81%)	22 (40.3%)	209 (40%)
Result status				
Pro‐cognitive	57 (19.6%)	23 (13%)	11 (21.1%)	90 (17.6%)
Negative	100 (33.7%)	20 (11%)	15 (28.8%)	135 (26%)
Dys‐cognitive	4 (1.3%)	—	—	4 (0.6%)
Not available/pending	131 (45%)	126 (74.4%)	26 (50%)	282 (55.2%)

**TABLE 2:** Drug trials with positive pro‐cognitive results.

Trial ID	Drug/dose/duration	Mechanism	Phase	Study design	Enrollment	Country	Sponsor	Duration	Cognitive test results
Glutamate modulators									
NCT02832037 [22]	BI 425809 (Iclepertin) 2, 5, 10, or 25 mg × 12 weeks	GlyT1‐inhibitor	2	DBRCT/parallel	509	Multi‐center	Boehringer Ingelheim	2018–2019	Greater improvements from baseline in the mean MCCB overall composite T‐score at Week 12 by Iclepertin 10 mg (*p* = 0.0122) and 25 mg (*p* = 0.0287)
NCT01047592 [23]	Sarcosine 2 g/d + benzoate 1 g/d × 12 weeks	GlyT1‐inhibitor	2	QBRCT/Parallel	63	Taiwan	Chang‐Hua Hospital	2009–2013	Improved global composite cognition (*p* = 0.03), neurocognitive composite score (*p* = 0.03), verbal learning and memory (*p* = 0.04).
NCT03382639 [24]	Luvadaxistat 50 mg/d × 12 weeks	DAAO‐inhibitor	2	DBRCT/Parallel	256	Multi‐centers	Neurocrine Biosciences	2018–2021	Improvement in BACS composite score (*p* = 0.031) and SCoRS interviewer total score (*p* = 0.011).
NCT00960219 [25]	Sodium benzoate 1 g/d × 6 weeks	DAAO‐inhibitor	2	QBRCT/Parallel	52	Taiwan	China Medical University Hospital	2009–2011	Improvement in MCCB composite (*p* = 0.4) with effect size of 0.67. Main improvement in processing speed (*p* = 0.03, *d* 0.065) and visual learning/memory (*p* = 0.02, *d* = 0.7)
NCT01116830 [26]	d‐serine, 60 mg/kg/d × 6 weeks	NMDA‐glycine agonist/DAAO inhibitor	2	DBRCT + Open label	66	USA	Hoffmann‐La Roche	2006–2009	Non‐significant and moderate effect size improvements were seen in verbal memory and attention/vigilance domains of MCCB with no significant change from baseline in MCCB composite.
NCT01474395 [27]	d‐serine 60 mg/kg 30 min prior to auditory plasticity	NMDA‐glycine agonist	2	QBRCT + open label/crossover	21	USA	Nathan Kline Institute for Psychiatric Research	2012–2013	Repeated administration of d‐serine led to improvements in perceptual organization & working memory indexes, phonological reading and auditory emotion recognition (*p* < 0.001).
NCT00455702 [28]	d‐cycloserine 50 mg/week × 8 weeks	NMDA‐glycine agonist	4	QBRCT/Parallel	38	USA	Massachusetts General Hospital	2006–2010	Improvement in Delayed thematic recall on the Logical Memory Test (*p* = 0.01). No change in MCCB composite.
NCT02769936 [29]	d‐cycloserine 100 mg single dose	NMDA‐glycine agonist	1	DBRCT/parallel	45	USA	University of California	2013–2015	Superior performance in working memory (*p* = 0.045) a day after in 2‐back task
NCT00733057 [30]	Minocycline, 200 mg/d × 6 months	Tetracycline antibiotic	3	TBRCT/parallel	54	Israel	Shalvata Mental Health Center	2003–2007	Reported a beneficial effect was found for cognitive functioning, mainly in executive functions (details were not available)
NCT01433055 [31]	Minocycline up to 200 mg d × 6 months	Tetracycline antibiotic	N/A	DBRCT/parallel	52	USA	University of Maryland, Baltimore	2011–2014	Global MCCB did not differ. There was a significant variation in size of treatment effects with improvement in working memory (*p* = 0.023)
NCT01493622 [32]	Minocycline 200 mg/d × 16 weeks	Tetracycline antibiotic	4	DBRCT/parallel	92	China	Central South University	2010–2011	Improvement in attention domain (*p* = 0.044) but no significant difference for other cognitive domains (*p* > 0.05).
NCT00757978 [33]	Memantine 20 mg/d + clozapine	NMDA antagonist	4	QBRCT/Parallel	22	Brazil	Hospital de Clinicas de Porto Alegre	2005–2008	Reported improvement in MMSE
IRCT20190606043827N1 [34]	Memantine 5 mg/d × 12 weeks + folic acid	NMDA antagonist	1	QBRCT/Parallel	50	Iran	Kashan University of Medical Sciences	2020‐N/A	The WAIS‐III score in the 12th week of the study was significantly different between the placebo and intervention groups (*p* = 0.004)
Nicotinic acetylcholine receptors (nAChRs) modulators									
NCT01655680 [35]	ABT‐126, 50 mg or 75 mg/×12 weeks	nAChR‐α7‐agonist	2	TBRCT/Parallel	432	USA	AbbVie (prior sponsor, Abbott)	2012–2014	No significant change at 12 weeks in MCCB. A significant difference in SCoRS total score compared with placebo at week 22 for ABT‐126 50 mg in the primary set (*p* = 0.004), and a trend was noted in the global rating scale (*p* = 0.054)
NCT01095562 [36]	ABT‐126 10 mg or 25 mg × 12 weeks	nAChR‐α7‐agonist	2	QBRCT/Parallel	207	USA	AbbVie (prior sponsor, Abbott)	2010–2011	No significant improvement in smoker; however, improvement in MCCB in non‐smoker (10 mg *p* = 0.021) and 25 mg (*p* = 0.001) and improvement in verbal learning (*p* = 0.003), working memory (*p* = 0.003) and attention (*p* < 0.001)
NCT01556763 [37]	EVP‐6124, of either 0.3 mg or 1.0 mg × 21 days	nAChR‐α7‐agonist	1	TBRCT/Parallel	21	USA	FORUM Pharmaceuticals Inc	2008–2008	CogState: Positive effects were found in performance on cognitive tests that measured non‐verbal learning, memory, executive function, and social cognition. There was a positive trend in total score (Day 1 *d* = 0.37, Day 21 *d* = 0.34 for total score).
NCT00968851 [38]	EVP‐6124 (Encenicline) 0.27 or 0.9 mg/d × 12 weeks	nAChR‐α7‐agonist	2	QBRCT/Parallel	317	Multi‐centers	FORUM Pharmaceuticals Inc	2009–2011	The LS mean difference for encenicline 0.27 mg was significant (Cohen's *d* = 0.257; *p* = 0.034) for the OCI score, Mean SCoRS total scores decreased showing improvement in function over time, and the difference was significant for encenicline 0.9 mg vs placebo (*p* = 0.011).
NCT00100165 [39]	DMXB‐A, 75 mg bid or 150 mg bid × 4 weeks	nAChR‐α7‐agonist	2	TBRCT/Parallel	29	USA	University of Colorado, Denver	2007–2009	No change in total MATRICS. The attention/vigilance domain T score significantly increased with DMXB‐A at both 75 mg b.i.d. (mean = 6.1, SD = 9.4) and 150 mg b.i.d. (mean = 7.6, SD = 10.6). The working memory domain T score showed a significant increase with 75 mg b.i.d. of DMXB‐A (mean = 4.6, SD = 6.5) and a nearly significant increase with 150 mg b.i.d. (mean = 4.5, SD = 7.3).
NCT01003379 [40]	TC‐5619, 1–25 mg × 8–12 weeks	nAChR‐α7‐agonist	2	QBRCT/Parallel/Quadruple	184	Multicenter	Targacept Inc.	2009–2010	GMLT improved at Week 4 (*p* = 0.036), with a trend at Week 12 (*p* = 0.081). TMT‐A showed a significant benefit at Week 8 (*p* = 0.046). No significant effects on most other cognitive, processing speed, or social cognition measures.
NCT01186471 [41]	Nicotine spray 0 mg, 1 mg or 2 mg × 3 times in separate sessions	nAChR agonist	1	QBRCT/crossover	32	Belgium	Janssen Pharmaceutica N.V., Belgium	2010–2012	Nicotine (1 mg) improved fairness‐sensitive rejections (measure of social cognition) in non‐smoker but no effect on smokers (*p* = 0.017); however, 2 mg dose loses its effect
NCT00383747 [42]	Transdermal nicotine patch	nAChR agonist	4	TBRCT/Parallel/	60	USA	Massachusetts General Hospital	2004–2009	Nicotine improved performance in both groups by reducing hit reaction time (*p* < 0.0001), its SD (*p* < 0.006), and random errors (*p* < 0.001). It led to a greater decrease in commission errors in schizophrenia vs. controls, with significant time × treatment × diagnosis interactions for false alarms (*p* < 0.01) and random errors (*p* < 0.02)
NCT01315002 [43]	Transdermal nicotine patch 7 mg in non‐smoker & 14 mg in smoker × 1 week	nAChR agonist	N/A	DBRCT/Crossover	48	Germany	University Hospital, Bonn	2008–2012	Nicotine significantly reduced antisaccade error rate in the standard trials, but not in the delayed trials (*p* = 0.02). Smoking status did not influence the nicotine effect on antisaccade error rate (*p* = 0.10) indicating an equal pro‐cognitive effect of nicotine in smokers and non‐smokers
Nicotinic acetylcholine receptors (nAChRs) modulators									
NCT00523445 [44]	Varenicline 0.5 mg/d for days 1–3, 0.5 mg BID for days 4–7, then 1 mg BID through week 8	nAChR‐α4β2‐partial agonist	3	DBRCT/Parallel	120	Korea	Inje University	2007–2009	No significant differences at end point of 8 weeks. In secondary longitudinal analyses, there were improvement in Digital Symbol Substitution Test (*p* = 0.013) and the WCST non‐perseverative errors (*p* = 0.043). Some treatment effects were different between smokers and non‐smokers.
NCT00492349 [45]	Varenicline 0.5 mg po qd × 7 days then titrated to Varenicline 0.5 mg po bid × 7 weeks	nAChR‐α4β2‐partial agonist	4	QBRCT/Parallel	69	USA	University of Maryland, Baltimore	2007–2016	A moderate dose of varenicline, significantly reduced the P50 sensory gating deficit in nonsmokers after long‐term treatment (*p* = 0.006), reduced startle reactivity (*p* = 0.02) regardless of baseline smoking status, and improved executive function by reducing the antisaccadic error rate (*p* = 0.03) regardless of smoking status. No significant effect on spatial working memory, predictive and maintenance pursuit measures, processing speed, or sustained attention by CCPT.
NCT00548470 [46]	Varenicline 0.5–1 mg/day in week 1, 2 mg/day (1 mg bid) in weeks 2–9.	nAChR‐α4β2‐partial agonist	4	Open label	14	USA	Nathan Kline Institute for Psychiatric Research	2007–2010	Reported significant improvements in scores, primarily associated with verbal learning and memory subscales of RBANS.
5‐Hydroxytryptamine (5‐HT) receptors modulators									
NCT01822418 [47]	Agomelatine 25 to 50 mg/d × 3 months	Antidepressant, 5‐HT2C antagonist/MT1 &MT2 agonist	4	Open label	27	Germany	Central Institute of Mental Health, Mannheim	2013–2015	Statistically significant yet clinically negligible increases of the MCCB composite score and the reasoning/problem solving sub‐score.
NCT01832285 [48]	Fluvoxamine 100 mg × 6 weeks	Antidepressant (SSRI & sigma‐1 receptor agonism)	N/A	Open label	29	Israel	Sha'ar Menashe Mental Health Center	2012–2013	Associative and logical verbal memory improved significantly and showed a significant correlation with changes in PMC, BDNF and GABA‐A beta3 receptor mRNA, which increased during treatment. Abstraction and object recognition improved, but this did not correlate with PMC measures
JPRN‐UMIN000003094 [49]	Fluvoxamine up to 150 mg/d × 12 weeks	Antidepressant (SSRI & sigma‐1 receptor agonism)	N/A	RCT/Parallel	48	Japan	Chiba University Graduate School of Medicine	2007–2010	The change from baseline to end point on the Spatial Working Memory strategy score (executive function) of CANTAB improved in the fluvoxamine group but no significant improvement in any of the subscales.
JPRN‐UMIN000006685 [50]	Tandospirone 30 mg/d × 6 weeks	Antidepressant, 5‐HT1A agonist	N/A	RCT/Parallel	26	Japan	University of Toyama	2011–2013	Significant time‐by‐group interaction effects were noted for the Wisconsin Card Sorting Test categories and WMS‐R verbal memory composite score.
5‐Hydroxytryptamine (5‐HT) receptors modulators									
NCT01602029 [51]	Ondansetron 8 mg/d + simvastatin 40 mg 6 months	Antiemetic, 5‐HT3 antagonist/Statin	2	DBRCT/Factorial	303	Pakistan	Pakistan Institute of Living and Learning	2010–2013	Improvement in Coughlan Verbal learning and Verbal Fluency test. No change in Stroop and WAIS Block design memory.
NCT00435370 [52]	Tropisetron 5 or 10 mg or 20 mg/d × 12 weeks	Antiemetic, 5‐HT3 antagonist	3	DBRCT/Parallel	40	USA/China	Baylor College of Medicine	2006–2011	RBANS: Improvement in immediate memory and total score. Delayed memory not significant but improved. No change in attention, visuospatial and language sub‐scores
JPRN‐UMIN000003084 [53]	Tropisetron 10 mg/day × 8 weeks	Antiemetic, 5‐HT3 antagonist	N/A	RCT/Parallel	40	Japan	Chiba University School of Medicine	2010–2009	The score on the rapid visual information processing (sustained visual attention) task of CANTAB.
Dopamine modulators									
NCT01519557 [54]	DAR‐0100A, 15 or 0.5 mg IV over 30 min × 5 days, repeat after 9 days	Dopamine‐1 agonist	Eary‐1	QBRCT/Parallel	68	USA	New York State Psychiatric Institute	2011–2013	Moderate improvement was detected on the CogState battery and on the attention domain of the MATRICS. No significant change in total MATRICS.
NCT00314639 [55]	Modafinil up to 200 mg/d × 70 days	Dopamine modulator	1/2	DBRCT/crossover	30	Canada	Laval University	2005–2009	A significant and rapid improvement was reported, particularly on attention and verbal fluency.
Acetylcholinesterase inhibitors (AchE‐inhibitor)									
NCT01490567 [56]	Donepezil 5 mg/d × 12 weeks	AchE‐inhibitor	4	QBRCT/Parallel	61	China	Central South University	2011–2012	At the 12‐week end point, the donepezil group showed significant improvements in the WMS Third Edition Spatial Span, Brief VMT total recall and delayed recall, TMT‐A, and CFT‐A (all *p* ≤ 0.018)
NCT00176423 NCT00077727 [57]	Galantamine 24 mg/d × 12 weeks	AchE‐inhibitor	4	QBRCT/parallel	86	USA	2002–2006	University of Maryland, Baltimore	Significant improvements on the WAIS‐III‐digit symbol and verbal memory measures
GABAergic modulators									
NCT00129441 [58]	Merck L‐830982, up to 8 mg bid × 4 weeks	GABA‐A‐α2 and α3 receptor agonists	2	TBRCT/Parallel	15	USA	University of Pittsburgh	2005–2008	Improved performance on the N‐back, AX‐CPT and memory index in RBANS.
NCT04822883	RL‐007, 10 mg or 20 or 40, or 80 mg TID × 8 days	GABAergic/glutamate and nAChRs modulator	2	Open label/sequential	2	USA	Recognify Life Sciences	2021–2022	Dose‐related improvements in BACS, Symbol Coding Test and HVLT, focusing on general cognitive function and episodic memory.
Oxytocin									
NCT01312272 [59]	Oxytocin, 40 IU intranasal, single dose	Oxytocin modulator	N/A	TBRCT/Parallel	23 M	USA	VA Greater Los Angeles Healthcare System	2011–2012	Significantly improved performance in high‐level social cognition (*p* = 0.045) with a large effect size (*d* = 1.00). Also, it reached a trend level of significance in improving the TASIT III sarcasm score. No significantly improve performance on the total social cognition composite measure.
NCT01517360 [60]	Oxytocin, 40 IU intranasal × twice weekly × 6 weeks (12 sessions) + social training	Oxytocin modulator	1	QBRCT/Parallel	27 M	USA	VA Greater Los Angeles Healthcare System	2012–2013	Significantly greater improvements in empathic accuracy (*p* = 0.03) at both posttreatment and 1 month follow up. No effects for the other social cognitive tests or neurocognition
ACTRN12609000528257 [61]	Oxytocin, 24 IU, intranasally, single dose	Oxytocin modulator	3	DBRCT/Crossover	22 M	Australia	University of Sydney	2009–2012	Enhanced performance on higher order social cognition tasks (Hinting Task F (1, 19) = 5.38, *p* = 0.03, and the non faux pas' condition of the Faux Pas Recognition Task *F* (1,19) = 6.37, *p* = 0.02), with no effects on general neurocognition
NCT01394471 [62]	Oxytocin 24 IU, bid × 12 weeks	Oxytocin modulator	1	QBRCT/single arm	68	USA	University of North Carolina	2011–2015	The study found no evidence for a differential advantage of oxytocin over placebo on social cognition. Among secondary outcomes, there was a modest advantage for oxytocin over placebo on a single component (SSPA Verbal SS *p* = 0.005)
NCT01568528 [63]	Oxytocin, 24 IU, intranasally, single dose	Oxytocin modulator	2	DBRCT/Parallel	20 M	USA	Emory University	2013–2017	Intranasal oxytocin enhanced the recognition of emotions during an emotion‐based ball‐tossing game
Estrogen receptor modulators									
NCT03043820 [64]	Raloxifene, 120 mg/d × 12 weeks	Estrogen Receptor Modulation	3	DBRCT/Parallel	102	Netherlands	Iris Sommer	2016–2021	Improvement of working memory sub score of BACS at week 38 (LSM 0.73; adjusted *p* = 0.040), while having negative effects on working memory at week 38 in men (LSM −0.53; adjusted *p* = 0.026).
ACTRN12608000461392 [65]	Raloxifene, 120 mg/d × 6 weeks	Estrogen Receptor Modulation	N/A	DBRCT/Crossover	98	Australia	University of New South Wales	2008‐N/A	Significant improvement relative to placebo in memory and attention/processing speed shown in LMI and LMII of the WMS‐R as well as in form A of the TMT‐A (*p* < 0.001).
IRCT2016010225812N1 [66]	Raloxifene 60 mg/d or isradipine 5 mg BID or placebo × 6weeks	Estrogen Receptor Modulation	2	DBRCT/Parallel	60	Iran	Chancellor for Research of Zanjan University of Medical Sciences	2017‐N/A	An association between adjunctive raloxifene treatment and the alleviation of verbal memory deficits in LM‐I as well as significantly improved the verbal memory and attention dysfunction in some variables of the Stroop test by Isradipine treatment. No effect was observed in processing speed and executive function deficits
Others									
ChiCTR1900021078 [67]	Betahistine 72 mg/d × 12 weeks	H1‐receptor agonist and H3‐receptor antagonist	N/A	DBRCT/Parallel	89	China	Beijing huilongguan hospital	2019–2020	Improvements in the MCCB composite score after 12 weeks of treatment (*p* = 0.003) as well as improvements in MCCB verbal learning (*p* = 0.02) and visual learning (*p* = 0.001) scores
NCT00690274	BF2.649 (Pitolisant) up to 20 mg/d × 12 weeks	H3‐receptor inverse agonist	2	DBRCT/Parallel	52	USA	University of Texas Southwestern Medical Center	2008–2018	The Brief Visuospatial Memory Test‐Revised (BVMT‐R) assesses visual memory. Participants receiving BF2.649 scored ~3.2 points higher on average than placebo. No *p* value or effect size available
NCT02006628 [68]	Cannabidiol (GWP42003), 1000 mg CBD/d × 6 weeks	Cannabinoid	2	DBRCT/Parallel	43	Multi‐centers	Jazz Pharmaceuticals	2014–2015	Greater improvements that fell short of statistical significance in cognitive performance (BACS: treatment difference = 1.31, 95% CI = −0.10, 2.72)
NCT03271866 [69]	Metformin 1500 mg/d × 12 weeks	Antihyperglycemic	A	Open‐labeled	72	China	Central South University	2017–2023	Improvements in the MCCB composite score, speed of processing, working memory, verbal learning, and visual learning
NCT01354132 [70]	NAC, 1800 AM × 6 months	TBRCT/parallel	N/A	USA	20	Israel	Beth Israel Deaconess Medical Center	2011–2014	Improved processing speed in both the verbal fluency (*p* = 0.023) and the TMT‐A (*p* = 0.048) tests, and in Processing Speed Factor (combining the above tasks) (*p* = 0.022).
IRCT20190914044769N1 [71]	Pentoxifylline 400 mg TID × 8 weeks	Vasodilator‐PDE‐4/PDE‐5 inhibitor	3	DBRCT/parallel	52	Iran	2020–2021	Mashhad University of Medical Sciences	The number of errors in the incongruent Stroop test, and reaction time in the congruent Stroop test were significantly lower in the treatment group (*p* < 0.05). The number of categories of WCST was significantly higher in the treatment group (*p* < 0.05).
NCT02079844 [72]	Roflumilast 100 or 250 μg/d × 8 days	PDE4 inhibitor	1	DBRCT/crossover	15	UK	2014–2015	Takeda	MCCB; verbal memory was significantly improved under 250 μg roflumilast (ES) = 0.77). Working memory was not improved (ES = 0.03).
NCT00514449 [73]	Valacyclovir 1.5 mg BID × 18 weeks	Antiviral	2	RCT/parallel	21	USA	2007–2016	Konasale Prasad	The PCNB showed improvement in verbal memory (*p* = 0.036), working memory (2‐back) (*p* = 0.3) and delayed visual memory (*p* = 0.026).
NCT01696929 [74]	Tocilizumab 4 mg/kg baseline and 4 weeks after	Monoclonal antibody	1	SINGLE_GROUP	8	USA	2012–2015	Brian Miller	Significant gains were observed in verbal fluency, digit symbol coding, and composite cognitive scores (using the BACS). *P* value were not reported in the published results.

Abbreviations: AM, morning dose; AX‐CPT, AX‐continuous performance test; BACS, brief assessment of cognition in schizophrenia; BDNF, brain‐derived neurotrophic factor; BID, twice a day; CANTAB, Cambridge neuropsychological test automated battery; CCPT, Conners' continuous performance test; CFT‐A, category fluency test‐animal naming; CI, confidence interval; CogState, CogState cognitive test battery; D, day; DAAO, d‐amino acid oxidase; DBRCT, double‐blind randomized controlled trial; DMXB‐A, 3‐(2,4‐dimethoxybenzylidene)‐anabaseine; ES, effect size; g, gram; GABA, gamma‐aminobutyric acid; GlyT1, glycine transporter type; GMLT, groton maze learning test; HVLT, hopkins verbal learning test; IU, international unit; LM‐I, logical memory I; LSM, least squares mean; MATRICS, measurement and treatment research to improve cognition in schizophrenia; MCCB, MATRICS consensus cognitive battery; Mg, milligrams; Min, minutes; MMSE, mini‐mental state examination; Mo, month; Mt., melatonin receptor; N/A, not applicable; NAC, *N*‐acetylcysteine; NMDA, *N*‐methyl‐d‐aspartate; OCI, overall cognitive index; PCNB, penn computerized neurocognitive battery; PMC, peripheral mononuclear cells; QBRCT, quadruple‐blind randomized controlled trial; RBANS, repeatable battery for the assessment of neuropsychological status; RCT, randomized controlled trial; SCoRS, schizophrenia cognition rating scale; SD, standard deviation; SSPA, social skills performance assessment; SSRI, selective serotonin reuptake inhibitor; TASIT, the awareness of social inference test; TBRCT, triple‐blind randomized controlled trial; TID, three times a day; TMT, trail making test; VMT, visuospatial memory test; WAIS, Wechsler adult intelligence scale; WCST, Wisconsin card sorting test; Wk(s), week (s); WMS‐R, Wechsler memory scale—revised.

### 4.2 Characteristics of Pharmacological‐Based Trials

From 290 selected drug studies, 57 (19.6%) reported pro‐cognitive properties in SSD, with one study reporting pro‐cognitive results in women and worsening cognitive test results in men (NCT03043820). Four trials showed dys‐cognitive results (NCT00455650, NCT00772005, NCT01077700, NCT01555697). Negative results were reported in 100 (33.7%) trials, and there were no results posted or found online for the remaining studies. Table 2 provides more details regarding studies with pro‐cognitive results. Table 3, supplemental materials, provides a summary of the primary compounds utilized in these trials, as well as the principal findings within each category. Figure 1, supplemental materials, presents the frequency of cognitive tests administered in pro‐cognitive trials.

**TABLE 3:** Device (neuromodulation) trials with pro‐cognitive evidence.

Trial ID	Intervention	Protocol	Study design	Enrollment	Country	Sponsor	Duration	Cognitive test results
NCT01494623 [75]	rTMS vs. sham	20 Hz, right and left DLPFC, 5 days/week for 20 sessions	DBRCT/parallel	27	Canada	Centre for Addiction and Mental Health	2006–2012	Significant improvement in working memory shown in 3‐back accuracy for targets compared with placebo sham (Cohen's *d* = 0.92)
NL‐OMON2519 [76]	rTMS vs. sham	10 Hz, bilateral DLPFC, 15 days	DBRCT/parallel	32	Netherland	University Medical Center Groningen	2009–2013	Semantic verbal fluency improved significantly (*p* = 0.006). No change in other cognitive tests.
ChiCTR1900024422 [77]	rTMS + family intervention × 12 weeks	10 Hz, left DLPFC, 5 times a week × 20 sessions	RCT/cross‐sectional	250	China	Zhengzhou University	2016–2017	Improved MCCB scores after 12 weeks in all groups and more in combined group
NCT04055181 [78]	rTMS vs. sham	10 Hz, left DLPFC, for 5 days a week × 4 weeks (20 sessions)	TBRCT/parallel	52	China	Beijing HuiLongGuan Hospital	2018–2020	Significant improvements in RBANS scores and SCWT cards 1 and 3 (all *p* < 0.05) were observed after 4 weeks in both active and sham rTMS groups. The active group showed greater gains in immediate (*p* = 0.016) and delayed memory (*p* = 0.047)
NCT02131129 [79]	rTMS vs. sham	20 Hz, bilateral, sequential DLPFC, 5 days/week × 2 weeks (10 sessions)	QBRCT/parallel	20	USA	Indiana University	2014–2016	The rTMS group showed greater improvement in BACS Composite Score at 2‐week follow‐up vs. sham (*p* = 0.018), but not at treatment end. Semantic & Letter Fluency (*p* = 0.014), Symbol Coding (*p* = 0.039), and Token Motor Task (*p* = 0.035) also improved.
NCT03273439 [80]	rTMS vs. sham	10 Hz, left DLPFC, for 5 days a week × 4 weeks	RCT/parallel/single	47	China	Suzhou Psychiatric Hospital	2012–2015	CANTAB; PRM performance metrics (percent correct and number correct) and changes in these metrics from baseline were significantly greater in the active rTMS group at week 8 compared to the sham group (all *p* < 0.05)
NCT03774927 [81]	rTMS vs. sham	20 Hz or 10 Hz, left DLPFC, 20 min for 5 days a week × 8 weeks	DBRCT/parallel	120	China	Beijing HuiLongGuan Hospital	2017–2018	BRANS: 20 Hz rTMS treatment produced therapeutic benefit on immediate memory of patients with chronic SCZ at week 8, but not in the 10 Hz group. Both 10 Hz and 20 Hz rTMS treatments produced delayed effects on cognitive functions at the 6‐month follow‐up
ChiCTR2300073220 [82]	rTMS vs. sham	20 Hz, left DLPFC, 5 times a week × 20 sessions	RCT/parallel	128	China	Shanghai Putuo Mental Health Center	2019–2020	Improvement in total scores of RBANS & SCWT; after 4 weeks with more prominent results in immediate memory, attention and language which sustained × 6 months. The effect on delayed memory was not sustained after 6 months.
NCT03288779 [83]	iTBS	Left DLPFC, 9 min, one session	Open label	6	USA	Duke University	2017–2018	3 subjects completed the treatment. One subject showed improvement in all domains of BACS, second subjects in 3 domains and third subject only in executive function.
NCT02128919 [84]	tDCS vs. sham	2 mA, left DLPFC, 20 min two times per day for 5 days	TBRCT/parallel	37	USA	Nathan Kline Institute for Psychiatric Research	2012–2016	Significant improvements in working memory (*p* = 0.002) and attention‐vigilance (*p* = 0.027) as measured by the MCCB. The composite MCCB score improved (*p* = 0.008).
NCT04184830 [85]	tDCS + cognitive training vs. sham	8 cognitive training sessions + 14 tDCS; 2 mA, Left mPFC, 30 min	SBRCT/parallel	49	UK	King's College London	2011–2015	No immediate effects of tDCS during stimulation but significant improvements in learning at 24 h (Session 3, *p* = 0.046) and at Day 56 (Session 7, *p* = 0.040).
NCT01607840 [86]	tDCS	Left and right DLPFC for 30 min × 2 sessions	SBRCT/crossover	11	USA	Johns Hopkins University	2012–2014	Anodal stimulation over the left DLPFC improved performance relative to cathodal stimulation on measures of working memory and aspects of verbal fluency relevant to word retrieval
NCT03485131 [87]	tDSC	2 mA, left DLPFC, 20 min, BID, 3 h × 4 weeks	TBRCT/parallel	28	USA	Manhattan Psychiatric Center	2014–2018	A small but significant improvement in working memory was observed in the tDCS group (*p* = 0.048), but no overall cognitive benefits were noted
NCT02748083 [88]	tDCS vs. sham	2 mA, left DLPFC, 20 min BID × 10 session	QBRCT/parallel	45	China	Shanghai Mental Health Center	2015–2017	No immediate post treatment results. Significant improvement in processing speed (MATRICS) (P‐0.00194 and Cohens' *d* 4.7) and accuracy in a 1‐back working memory task (Costate).
UMIN000015953 [89]	tDCS	2 mA, left DLPFC, 20 min BID per day for 5 days	Open label	28	Japan	National Center of Neurology and Psychiatry	2015–2017	Significant improvement in BACS composite scores (*d* = 0.49). Largest effects in verbal memory (*d* = 0.55), followed by motor/speed (*d* = 0.44) and verbal fluency (*d* = 0.36).
IRCT20180317039116N1 [90]	tDCS + OT vs. sham	2 mA intensity, DLPFC for 20 min × 12 sessions	RCT/parallel	24	Iran	University of social welfare and rehabilitation sciences	2018‐n/a	Significant improvements in SRM (*p* = 0.016, *ηp* ^2^ = 0.295). Paired‐Associate Learning (PAL) (*p* = 0.037) & Attention (*p* = 0.013).
JRCTs032180026 [91]	tDCS	2 mA, left STS and for 20 min BID for 5 days	Open label	15	Japan	Sumiyoshi Tomiki	2018–2021	Significant improvements were found on theory of mind, as measured using the SCSQ (*d* = 0.53) and the HT (*d* = 0.49). No improvement in BACS
NCT04870996 [92]	tDCS + cognitive training vs. sham + cognitive training	2 mA × left DLPC, 20 min × 5 session	DBRCT/parallel	44	Hong Kong	Chan Sau man, Sandara	2019–2020	Significant time effects (*p* < 0.001), and “group‐by‐time” interaction effect (*p* = 0.036) in the BDST which represents the cognitive domain of verbal working memory and sustained in 1‐month post‐intervention.
NCT06538259 [93]	tDCS + retrieval practice vs. sham	2 mA × left DLPC, 2 sessions/day 20 min × 5 days + word practice	DBRCT/parallel	52	China	National Natural Science Foundation of China	2023–2025	Using MOCA as cognitive test, anodal tDCS improved recall (*p* = 0.008) and organization (*p* = 0.011), while retrieval practice outperformed restudy (*p* < 0.001); effects were strongest when combined (*p* = 0.040, *p* = 0.007)
NCT04545294 [94]	tACS + VM task vs. sham + VM taks	6 Hz, 2 mA, frontoparietal × 20 min × 5 consecutive days	DBRCT/parallel	36	Taiwan	Tri‐Service General Hospital	2019–2020	BCIS showed efficacies for cognitive symptoms, Working memory capacity (*p* < 0.05)
NCT03872310 [95]	tACS vs. sham	6 Hz, 1.5 mA right DLPFC & right FPC, 15 min	DBRCT/crossover	28	Taiwan	Taipei Medical University Shuang Ho Hospital	2019–2021	Right frontoparietal theta tACS significantly improved accuracy in low‐performers (*p* = 0.038, *ηp* ^2^ = 0.243) but had no effect on high‐performers.
NCT04620460 [96]	rTUS vs. sham	500 Hz, LIFUS, Left DLPFC, 1 session/d × 15 days	DBRCT/parallel	32	China	Shanghai Mental Health Center	2020–2022	CPT scores improved significantly (*p* = 0.05), suggesting an enhancement in attention and executive function.

Abbreviations: BACS, brief assessment of cognition in schizophrenia; BCIS, beck cognitive insight scale; BDST, backward digit span test; BID, twice a day; CANTAB, Cambridge neuropsychological test automated battery; CPT, continuous performance test; DBRCT, double‐blind randomized controlled trial; FPC, frontoparietal cortex; Hr(s), hour(s); HT, hinting task; iTBS, intermittent theta‐burst stimulation; LIFUS, low‐intensity focused ultrasound stimulation; MATRICS, measurement and treatment research to improve cognition in schizophrenia; MCCB, MATRICS consensus cognitive battery; PRM, pattern recognition memory; QBRCT, quadruple‐blind randomized controlled trial; RBANS, repeatable battery for the assessment of neuropsychological status; RCT, randomized controlled trial; rTMS, repetitive transcranial magnetic stimulation; rTUS, repetitive transcranial ultrasound; SBRCT, single‐blind randomized controlled trial; SCSQ, social cognition screening questionnaire; SCWT, stroop color and word test; SCZ, schizophrenia; SRM, spatial recognition memory; tACS, transcranial alternating current stimulation; TBRCT, triple‐blind randomized controlled trial; tDCS, transcranial direct current stimulation; Wk(s), week(s).

#### 4.2.1 Glutamate Modulators

Nearly one fourth of trials (*n* = 63) investigated medications which modulate the glutamate system, mainly through enhancing the function of the glycine site of *N*‐Methyl‐d‐Aspartate (NMDA) receptors either directly or indirectly. Among these molecules, sarcosine (in some trials considered a dietary supplement) which acts as a glycine transporter type 1 inhibitor showed benefits in improving global neurocognitive composite score (*p* = 0.03) in addition to verbal learning and memory (*p* = 0.04; [23]). More recently, BI425809 (Iclepertin), an investigational selective inhibitor of the glycine transporter 1, was developed specifically to target cognitive impairment associated with schizophrenia. Both Iclepertin 10 (*p* = 0.01) and 25 mg (*p* = 0.01) for 12 weeks demonstrated improvement in total MATRICS Consensus Cognitive Battery (MCCB) composite scores in a multicenter, phase 2, randomized clinical trial with 509 participants, and at the time of initial data extraction had an active phase 3 trial [22]; unfortunately, the compound developer announced on January 16, 2025, that the results of the phase 3 trials did not meet primary end points.

Luvadaxistat is a d‐amino acid oxidase (DAAO) inhibitor which was designed to improve negative symptoms in schizophrenia. However, the trial assessed cognitive function as a secondary outcome and showed pro‐cognitive evidence in phase 2 with an improvement in Brief Assessment of Cognition in Schizophrenia (BACS) composite score (*p* = 0.03) and Schizophrenia Cognition Rating Scale interviewer total scores (*p* = 0.01; [24]). As the study did not meet its primary outcome to improve negative symptoms, the manufacturer halted further development as of September 2024. Sodium benzoate is another DAAO inhibitor that showed improvement in processing speed (*p* = 0.3), visual learning and memory (*p* = 0.2) and MCCB composite score (*p* = 0.4) in one study [25].

There have been other trials that showed partial pro‐cognitive results for glutamate modulators, by improving sub‐scores of cognitive batteries. In particular, d‐serine, an endogenous amino acid and co‐agonist of the glycine site of NMDA receptor [26, 27], and d‐cycloserine, an antibiotic with additional partial agonism function at the glycine site of NMDA receptors [28, 29] showed improvement in working and verbal memory. Although minocycline, a broad‐spectrum tetracycline antibiotic, did not improve composite scores, it did show positive results in improving working memory and attention in 3 moderate‐sized, double‐blind randomized clinical trials (DBRCT). The mechanism of action of minocycline is perceived to be through modulation of the glutamate system [30, 31, 32]. Memantine, a non‐competitive NMDA receptor antagonist approved by the FDA for moderate to severe Alzheimer's disease, showed pro‐cognitive properties in two small sample sizes but placebo‐controlled studies [33, 34]; however, in another trial it is mixed or detrimental compared to placebo (NCT01555697).

#### 4.2.2 Nicotinic Receptors Modulator

Another major category of investigational drugs (*n* = 32) belongs to modulators of nicotinic acetylcholine receptors (nAChRs). Several investigational drugs target enhancement of the α7 subunit of nAChRs. Although ABT‐126, a potent and selective agonist of α7 nAChRs, showed a significant difference in MCCB in non‐smokers in both 10 mg (*p* = 0.02) and 25 mg (*p* = 0.001) doses in early studies [36], its cognitive enhancing results remained inconsistent in the following study with 50 mg and 75 mg doses [35] as it did not improve MCCB parameters despite improving in SCoRS (Schizophrenia Cognition Rating Scale) total score compared with placebo at week 22 (*p* = 0.004).

Encenicline (EVP‐6124), another selective α7 nAChR, showed positive effects on neurocognitive measures, in visual memory, working memory, executive function, and social cognition in a phase I trial. A meaningful advantage for EVP‐6124 over placebo was observed in the total composite score of the CogState schizophrenia battery (Day 1 *d* = 0.37; Day 21 *d* = 0.34) [37]. Additionally, in a subsequent phase II study, Encenicline showed benefits in improving the Overall Cognitive Index (OCI) at dose of 0.27 mg with a small effect size and in SCoRS total score at dose of 0.9 mg (*p* = 0.01; [38]). Notably, the development of this compound ended in March 2016 as the global phase III trial did not meet the endpoints for cognitive impairment in schizophrenia.

DMXB‐A, a partial agonist of α7 nAChRs, showed effects on the attention/vigilance and working memory MATRICS domains [39]. Nevertheless, a subsequent phase II study published in 2018 showed no significant difference in these domains with DMXB‐A extended‐release formulation [97]. TC‐5619 (Bradanicline), another α7 nAChRs partial agonist, showed improvement in visual learning and memory in the Groton Maze Learning Task (GMLT) at Week 4 (*p* = 0.03), with a trend at Week 12 (*p* = 0.08) and processing speed in TMT (Trail Making Test) at Week 8 (*p* = 0.04; [40]). However, the subsequent phase IIb trial did not show any significant outcome [98].

In two randomized, placebo‐controlled trials, nicotine patches (nicotine being a non‐selective nAChR agonist) demonstrated improvement in parameters of cognition such as increased response bias indicating enhanced reward responsiveness [42] and reduced anti‐saccade errors (*p* = 0.02; [43]). In addition, varenicline, a partial α4β2‐nAChR agonist approved by the FDA for smoking cessation, showed benefits by improving different domains of cognition such as processing speed, working memory, and cognitive flexibility in 2 medium‐sized DBRCTs [44, 45] and in verbal learning and memory in an open‐label study [46]. In another study [41], nicotine spray (1 mg) improved fairness‐sensitive rejections (measure of social cognition) in non‐smokers, but there was no effect on smokers (*p* = 0.017).

Among modulators of nicotinic receptors, mecamylamine—a non‐selective nAChR antagonist originally developed as a hypertensive agent—was reported to worsen the measures of attention in patients with schizophrenia compared to varenicline and placebo [99].

### 4.3 Others

Among 5‐Hydroxytryptamine (5‐HT) receptor modulators, antidepressants such as agomelatine, a 5‐HT2C antagonist [47], fluvoxamine, a selective serotonin reuptake inhibitor [48, 49], and anxiolytics such as tandospirone, a 5‐HT1A agonist [50] exhibited benefits in some aspects of cognition in small‐scale trials. It is worth noting that buspirone, another azapirone derivative with 5‐HT1A partial agonist activity, has also been reported to improve certain features of cognitive function in two published studies [100, 101] but had negative results in another study [102]. However, the other buspirone‐related studies that were identified through our search (NCT00178971 and NCT06906224) have not reported results.

Antiemetics with 5‐HT3 antagonism properties such as ondansetron [51] and tropisetron [52, 53] improved aspects of verbal learning, memory, and visual information processing. Nonetheless, results remained inconsistent between trials.

Modafinil, a dopamine modulator approved by the FDA for the treatment of narcolepsy, has been shown to enhance attention and verbal fluency in a clinical trial [55]. In another trial, an investigational dopamine‐1 receptor agonist, DAR‐0100A (dihydrexidine), showed moderate improvement in working memory [54]. Despite that, armodafinil performed poorly compared to placebo in another study [103].

Acetylcholinesterase inhibitors such as galantamine [57] and donepezil [56] which are primarily indicated in the management of mild to moderate Alzheimer's disease, were investigated in CIAS and have shown limited benefits in improving memory.

Merck L‐830982, a Gamma‐Aminobutyric Acid (GABA)ergic modulator, showed improvements in working memory, attention, and general cognitive ability in a small sample size phase 2 trial [58]. Another GABAergic modulator (also nicotinic and glutaminergic modulator), RL‐007 (N121CT04822883), was purported to show improvement in general cognitive function and episodic memory; however, no statistical details were released. The phase 2 trial's result of RL‐007 is pending https://ir.atai.life/news‐releases/news‐release‐details/atai‐life‐sciences‐initiates‐phase‐2b‐proof‐concept‐trial‐rl‐007.

Interestingly, oxytocin, an endogenous neuropeptide mainly known to be involved in uterus contraction during childbirth, showed improvements in components of social cognition batteries in a few RCTs. For example, intranasal oxytocin demonstrated improvement in detection of sarcasm and deception [59], empathy accuracy [60], recognizing social faux pas and indirect hint [61], social skill performance [62] and emotion recognition [63]. On the other hand, raloxifene, an estrogen receptor modulator, also exhibited pro‐cognitive evidence in 2 medium‐size studies, mainly in working and verbal memory [65, 66]; however, worsening cognition was noted in male subjects in one of the trials [64].

While betahistine, an H1 (Histamine) receptor agonist and H3 receptor antagonist ([67]) showed improvements in MCCB composite score after 12 weeks (*p* = 0.003), the results of other histamine modulator trials were inconsistent. For example, the results of one unpublished study (NCT00690274) investigating BF2.649 (Pitolisant) suggested improvement in the Brief Visuospatial Memory Test, a parameter of visual memory; however, the MCCB composite score was worse after treatment with ABT‐288, another H3 receptor antagonist, compared to placebo [104].

Other compounds with isolated positive results were metformin [69], valacyclovir [73], N‐acetylcysteine (NAC) [70], pentoxifylline [71], roflumilast [72], cannabidiol [68] and Tocilizumab [74].

### 4.4 Characteristics of Device‐Based Trials

Transcranial magnetic stimulation techniques, in particular, repetitive Transcranial Magnetic Stimulation (rTMS) (*n* = 47) and intermittent Theta Burst Stimulation (iTBS) (*n* = 15) collectively accounted for 47% of the neuromodulation trials. Nine of these trials demonstrated pro‐cognitive results, eight were negative, and the results for the remaining are not available yet. The most common protocol used in these trials was 10–20 Hz, targeting primarily the left dorsolateral prefrontal cortex (DLPFC) for 15–20 sessions. Improvement in global cognition, working memory, attention, and processing speed was noted in the positive trials (Table 3).

For example, rTMS showed improvement in attention, immediate memory, language, and overall cognitive function measured after 20 sessions of stimulation in one study in 2013 [75]. In a proof‐of‐principle study in 2015 that was investigating the effect of rTMS on brain activation in schizophrenia, semantic verbal fluency improved after rTMS treatment (*p* = 0.006), although there were no significant effects on other cognitive tests [76]. In a larger scale study in China, improvement in total MCCB was noted when rTMS was combined with family intervention; especially in attention/vigilance (*p* < 0.01), working memory (*p* = 0.008), and social cognition [77]. Improvement in immediate memory (*p* = 0.016) and delayed memory (*p* = 0.047) was also observed in patients with chronic schizophrenia [78]. Other studies showed sustained effects at 2 weeks [79], 8 weeks [80] and 6 months [81, 82] follow‐up with moderate effect size. In an open‐label very small study of iTBS (intermittent Theta Burst Stimulation), improvement in verbal memory and motor performance was reported [83]; the results of other iTBS studies are not available yet.

Electrical stimulation techniques such as Transcranial Direct Current Stimulation (tDCS) (*n* = 48) and transcranial Alternative Current Stimulation (tACS) (*n* = 14) were other major investigational neuromodulation techniques. Among the tDCS studies, 10 reported positive results and 10 were negative. The protocol often used was 2 mA intensity for 20–30 min per session over the left DLPFC and for a variable number of sessions.

The tDCS results indicated improvement in the overall composite of MCCB [84], learning and decision making [85], working memory [86, 87], processing speed [88], verbal memory [89, 92], recognition memory [90], and social cognition [91]. One study showed improvement in the Montreal Cognitive Assessment test [93]. Regarding tACS, the results were available for only two trials, which showed benefits in improving scores related to working memory [94, 95].

Low intensity Repetitive Transcranial Ultrasound Stimulation (rTUS), in an isolated DBRTC, showed improvement in continuous performance test, a parameter of attention [96].

One study that was investigating low‐level laser therapy reported negative results [105] and the results for the remaining trials investigating Transcutaneous Vagus Nerve Stimulation (*n* = 1), normobaric oxygen (*n* = 1), Transcranial Pulsed Electromagnetic Field Stimulation (*n* = 1), Modified Electroconvulsive Therapy (*n* = 1), Deep Brain Stimulation (*n* = 4) and acupuncture (*n* = 2) were not available at the time of this review.

### 4.5 Characteristics of Dietary‐Supplement Trials

Eleven (21.1%) studies reported positive results, 15 (28.8%) negative results, and 26 (50%) are pending results. Table 4 provides more details about positive trials.

**TABLE 4:** Dietary supplement trials with pro‐cognitive evidence.

Trial ID	Drug/dose/duration	Study design	Enrollment	Country	Sponsor	Duration	Cognitive test results
NCT00177177 [106]	l‐carnosine, 2 g/d × 3 months	QBRCT/parallel	75	USA	University of Pittsburgh	2004–2007	Faster reaction times in non‐reversal trials for L‐carnosine group (*p* = 0.005–0.05, small‐medium effect sizes). Improved strategic efficiency in the l‐carnosine group (*p* = 0.022). Reduced perseverative errors (*p* = 0.015, medium effect size) with odds ratio of 3.5 times.
NCT00996242 [107]	l‐lysine 6 g/d × 4 weeks	SBRCT/crossover	10	Sweden	Göteborg University	2007–2009	Performance on WCST (executive function & cognitive flexibility) improved: Fewer total errors (*p* = 0.024), fewer perseverative errors (*p* = 0.032), fewer perseverative answers (*p* = 0.026). It was noted that improvement might be influenced by practice effects.
NCT00615511 [108]	Pregnenolone 500 mg bid vs. placebo × 8 weeks	DBRCT	120	USA	Weill Medical College of Cornell University	2007–2014	No significant changes compared to placebo were demonstrated in composite MCCB scores. In contrast, pregnenolone significantly improved functional capacity (*p* = 0.03), particularly in communication skills (*p* < 0.001) in functional capacity scores (UPSA‐B).
NCT00847600 [109]	Pregnolone 50 mg/d × 8 days	DBRCT	60	Israel	Sha'ar Menashe Mental Health Center	2009–2010	PREG improved deficits in visual attention (Matching to Sample Visual Search task, *p* = 0.002, *d* = 0.42) with moderate effect size. Also, improvements in sustained attention (*p* = 0.038) and executive function tasks (*p* = 0.049 for Stockings of Cambridge, *p* < 0.001 for Spatial Working Memory) noted.
NCT01716858A [110]	Sulforaphane, 30 mg/d × 8 weeks	Open label	7	Japan	Chiba University	2012–2015	CogState; The mean score in the Accuracy component of the OCL increased significantly after the trial (*p* = 0.043). No other improvement.
NCT02880462 [111]	Sulforaphane, 6 tablets or 4 of/d × 22 weeks	QBRCT/parallel	172	China	Central South University	2016–2019	There were no significant effects MATRICS overall composite score. improvement in three cognitive domains; Spatial Working Memory (*p* = 0.004), Reasoning and Problem‐Solving (*p* = 0.063, trend), Verbal Learning (*p* = 0.031).
NCT03153046 [112]	B‐GOS (probiotic), one sachet/d × 6 months	DBRCT/crossover	39	UK	University of Oxford	2017–2018	The composite T‐score of BACS (effect size: Cohen's *d* = 0.443) and executive functions (*t* = −2.114, *p* = 0.045, *d* = 0.432) improved at 6, 12 months follow‐up and sustained at 24 months.
IRCT20211118053100N1 [113]	Probiotics + vitamin D (400 IU)/d × 12 weeks	DBRCT/parallel	70	Iran	University of social welfare and rehabilitation sciences	2021–2022	Increase of 1.96 points in MoCA scores (*p* = 0.004) and more patients in the intervention group achieved a MoCA score ≥ 26 (*p* = 0.031).
NCT01759485 [114]	Vitamin D3, 14,000 IU weekly × 8 weeks	QBRCT/parallel	47	Israel	Geha Mental Health Center	2014–2017	Improvement in subscales of attention (*p* = 0.026) and delayed recall (*p* = 0.049) of MoCA.
ChiCTR2300070391 [115]	Berberine 300 mg TID × 3 months	RCT/parallel	30	China	Kangci Hospital of Jiaxing	2020–2021	The TMT‐A score was significantly lower (improved performance) at month 2 (*p* = 0.041) & 3 (*p* = 0.021). The TMT‐B was lower but not significant at endpoint; however, it was significant in follow‐up (*p* = 0.037).

Abbreviations: BACS, brief assessment of cognition in schizophrenia; BID, twice a day; CogState, cognitive test battery; DBRCT, double‐blind randomized controlled trial; IU, international unit; MATRICS, measurement and treatment research to improve cognition in schizophrenia; MCCB, MATRICS consensus cognitive battery; MoCA, Montreal Cognitive Assessment; OCL, one card learning; PREG, pregnenolone; QBRCT, quadruple‐blind randomized controlled trial; SBRCT, single‐blind randomized controlled trial; SWM, spatial working memory; TID, three times a day; TMT, trail making test; UPSA‐B, UCSD performance‐based skills assessment.

l‐Carnosine, an antioxidant and anti‐glycation agent, hypothesized to improve executive function through enhancing faster reaction time in non‐reversal trials of the set‐shifting test (*p* = 0.005) and reducing perseverative errors (*p* = 0.01) with a small to medium effect size [106]. Another trial with pro‐cognitive results investigated l‐lysine, an amino acid, and showed improvement in elements of executive function and cognitive flexibility of the Wisconsin Card Sorting Test (WCST) [107]. In two double‐blind, placebo‐controlled RCTs, pregnenolone, a neurosteroid, demonstrated improvement in functional capacity (*p* = 0.03) and communication skills (*p* < 0.001) [108] as well as visual attention (*p* = 0.002) and executive function (*p* < 0.001) [109].

Sulforaphane (broccoli sprout extract), a glutathione modulator [116], was examined in one open‐label and one DBRCT. Improvement was observed in the accuracy component of the One Card Learning Task (OCLT) (*p* = 0.04) suggestive of enhancing recognition learning [110] as well as spatial working memory, verbal learning, and problem solving sub‐scores of MCCB [111].

Probiotics were another category of supplements that have been investigated. One study showed improvement on BACS total t‐score and in domains related to executive function (*p* = 0.04) after 6 months, which were sustained up to 24 months [112]. Two studies reported pro‐cognitive effects either with vitamin D alone (*p* = 0.02; [114]) or in combination with probiotics (*p* = 0.004; [113]), although Montreal Cognitive Assessment (MoCA) was used as the main cognitive battery. Finally, berberine (a key ingredient in certain traditional Chinese medicine remedies) showed enhancement in processing speed and executive function through improvement of TMT‐A and TMT‐B scores (*p* < 0.05; [115]).

## 5 Discussion

Overall, although standardized composite batteries such as MCCB, BACS, RBANS (Repeatable Battery for the Assessment of Neuropsychological Status) and CANTAB (Cambridge Neuropsychological Test Automated Battery) were used in many trials across different categories of interventions, only a few showed improvements in total composite scores (Table 4 supplemental materials) with most trials showing improvements only in sub‐domains of these tests. In addition, the tests used in most trials tend to assess cognitive competence (performance on structured neuropsychological tests) under controlled conditions rather than functional performance and how the results translate into real‐world abilities. Similarly, social cognition was often secondary or exploratory in trial design, with only a few trials using dedicated social cognition tasks.

In terms of pharmacological interventions targeting CIAS, although initial efforts centered on 5‐HT1A partial agonists such as tandospirone and buspirone [117], in recent years the focus has been largely on glutamate modulators, particularly glycine transporter‐1 inhibitors such as sarcosine and Iclepertin. While phase I and II trials suggested promising results with improvement in total MCCB score, in the case of Iclepertin, the phase III trial did not support the preliminary results. Similarly, other glutamate modulators showed domain‐specific improvements in memory or attention, but results have been inconsistent. Likewise, nicotinic acetylcholine receptor modulators, another major category that were investigated as cognitive enhancers in SSD, failed to demonstrate consistent results in larger trials.

Beyond these major investigational trials, serotonin receptor modulators such as agomelatine and fluvoxamine, 5‐HT3 antagonists including ondansetron and tropisetron, dopaminergic compounds such as modafinil and DAR‐0100A as well as acetylcholinesterase inhibitors like galantamine and donepezil generated limited pro‐cognitive effects. Among GABAergic modulators, Merck L‐830982 and RL‐007 exhibited positive results in small studies; however, the results of larger studies are not available.

Unconventional pharmacological approaches, including oxytocin and raloxifene, demonstrated benefits in elements of social cognition and working memory, respectively, though findings were inconsistent across sex. Additionally, other pharmacological agents including NAC, metformin, valacyclovir, pentoxifylline, roflumilast, cannabidiol, and betahistine each reported isolated positive results in small‐scale studies, but these require replication.

Notably, recent exploratory analyses of xanomeline/trospium (Cobenfy), a novel FDA‐approved medication for positive symptoms of schizophrenia with M1/M4 muscarinic receptor agonism, revealed improvements in verbal memory, sustained attention, and executive function in a cognitively impaired subgroup of SSD. The trials (*N* = 357) showed significant cognitive gains compared to placebo in the cognitively impaired subgroup, with baseline performance ≤ 1 SD below norms, with a medium effect size (*d* = 0.54) that increased to *d* = 0.80 for participants with more severe impairments defined as scores at or below 1.5 standard deviations below the norm [118]. This study was not captured in our search, as cognitive enhancement was not registered as a primary or secondary outcome in the study protocol (NCT04659161and NCT04738123). Nonetheless, this study shows the potential for novel pharmacological strategies to address cognitive impairment in schizophrenia, in selected patient subgroups.

Among device‐based interventions, repetitive transcranial magnetic stimulation (rTMS) and intermittent theta burst stimulation (iTBS) studies reported improvements in attention, working memory, and processing speed. While several trials reported positive results, the overall response rate has been moderate and inconsistent, with some studies yielding negative results. A recent umbrella review [119] concluded that high‐frequency rTMS (> 100% motor threshold) targeting the left dorsolateral prefrontal cortex for at least 3 weeks is likely the preferred protocol to improve negative symptoms and can enhance working memory, executive function, and language. However, no consistent or significant improvement in attention and processing speed was reported, and publication bias remains a concern as negative results tend not to be published.

One of the notable observations in rTMS studies is the reported long‐lasting cognitive benefits, up to 6 months in some trials, potentially due to neuroplastic changes induced by stimulation. A body of preclinical evidence supports the role of rTMS in mechanisms involved in neuroplasticity such as enhancing glutamatergic synaptic strength and structural remodeling of dendritic spines in mice [120], altering protein expression and histone acetylation in the rodent frontal cortex [121], improving synaptic plasticity and increasing brain‐derived neurotrophic factor (BDNF) [122] as well as modulating microglial cytokine release [123].

Other forms of neuromodulation, particularly tDCS and tACS, may have potential for cognitive enhancement in SSD. A meta‐analysis [124] suggested that tDCS has a positive effect on working memory in SSD, based on data from 270 participants across nine randomized controlled trials. As for tACS, two systematic reviews [125, 126] suggested that tACS may improve various domains of cognition in SSD, although the current evidence is limited and inconsistent. Additionally, preliminary results of one study suggested that rTUS may improve attention. It is hypothesized that rTUS can induce neuromodulation through different biological effects by targeting both cortical and deep brain targets [127] which can have various neuropsychiatric applications [128].

As for dietary supplements, overall, the evidence for improving CIAS is limited and inconsistent. While compounds such as l‐carnosine, pregnenolone, sulforaphane, and probiotics (with vitamin D) have shown domain‐specific benefits, the improvements are often modest, vary by study, and none have demonstrated generalized effects. Until supported by larger studies, supplements remain experimental in this context.

## 6 Conclusions

Significant research efforts have been initiated in the last 25 years to address cognitive impairment in schizophrenia spectrum disorders. Despite these substantial efforts and partial positive results, no single intervention demonstrated consistent, replicable pro‐cognitive efficacy in SSD, underscoring the challenges of targeting cognitive symptoms in this population.

Potential avenues to explore include refining patient selection criteria to address individual cognitive needs, longer follow‐ups as well as ecologically valid assessments of how cognitive functions are deployed in everyday life. With multiple ongoing trials and novel modalities, research on neuromodulation and cognitive function in schizophrenia remains an evolving field. Adopting integrative approaches and perhaps combining pharmacotherapy, neuromodulation, and cognitive remediation techniques based on current findings could be considered.

## Author Contributions

Study concept and design, literature review, data analysis and synthesis and presentation, primary draft of the manuscript: Bahareh Peyrovian. Manuscript review and revision: Lena Palaniyappan. Manuscript review and edit: Theodore T. Kolivakis. Study concept, manuscript review and supervision: Howard C. Margolese.

## Conflicts of Interest

Lena Palaniyappan's research is supported by the Canada First Research Excellence Fund, awarded to the Healthy Brains, Healthy Lives initiative at McGill University (through a New Investigator Supplement to LP) and Monique H. Bourgeois Chair in Developmental Disorders and the Graham Boeckh Foundation. He receives a salary award from the Fonds de recherche du Québec‐Santé (FRQS). Lena Palaniyappan also acknowledges Canada Foundation for Innovation (CFI)'s John R. Evans Leaders Fund (JELF), matched by the Government of Quebec (Studying the Neural Basis of Interpersonal Interactions in Severe Mental Illnesses #43849 to LP). Dr. Kolivakis has received honoraria, sponsorship or grants for his participation as an advisory board member and/or as a speaker at educational events for AbbVie, HLS Therapeutics, Johnson & Johnson, Lunbeck, and Otsuka, and has received research support from Newron. Dr. Margolese has received honoraria, sponsorship or grants for his participation as an advisory board member and/or as a speaker at educational events for AbbVie, HLS Therapeutics, Johnson & Johnson, Lunbeck, Otsuka, Newron, and Teva and has received research support from the MGH hospital Foundation, Aifred Health, Newron and Syneurx. The other authors declare no conflicts of interest. [Correction added on 16 September 2025, after first online publication: Conflicts of interest section has been updated.]

## Supporting information


**Data S1:** Supporting Information.

**Data S2:** Supporting Information.

**Table S1:** Supporting Information.

**Table S2:** Supporting Information.

## Data Availability

The data supporting the findings of this study were obtained from publicly accessible sources, specifically the WHO International Clinical Trials Registry Platform (https://www.who.int/tools/clinical‐trials‐registry‐platform) and ClinicalTrials.gov (https://clinicaltrials.gov). Extracted data were compiled into spreadsheets by the authors for analysis, and the collected datasets are available from the corresponding author upon reasonable request.
