# Supplementary material for: Pharmacological and Mechanistic Interventions for Cognitive Impairment Associated With Schizophrenia: A Review of Registered Clinical Trials
Source: Acta Psychiatr Scand. 2025 Sep 4;153(5):402–31. doi: 10.1111/acps.70034 (PMC13050601; doi:10.1111/acps.70034)
Supplement: Supplementary file 2 — Data S2: Supporting Information. [file ACPS-153-402-s002.docx]

**Supplemental material- Table 3**

| **Drug Group** | **Key Compounds** | **Notable Findings** |
| --- | --- | --- |
| **Glutamate Modulators** | Sarcosine, BI425809 (Iclepertin), D-serine, d-cycloserine, Minocycline, Memantine, Luvadaxistat | Sarcosine improved global neurocognitive composite score, working and verbal memory  BI425809 showed improvement in MCCB scores but failed in phase 3  D-serine and d-cycloserine improved working and verbal memory  Minocycline improved working memory and attention |
| **Nicotinic Receptor Modulators** | ABT-126, Encenicline, DMXB-A, TC-5619, Nicotine patch, Varenicline | ABT-126 showed improvement in SCoRS but not MCCB  Encenicline improved CogState battery scores in phase I/II but failed in phase III  Nicotine patch improved reward responsiveness and reduced antisaccade errors  Varenicline improved processing speed, working memory, and cognitive flexibility |
| **Serotonin (5-HT) Modulators** | Agomelatine, Fluvoxamine, Tandospirone, Ondansetron, Tropisetron | Showed benefits in some aspects of cognition, but results were inconsistent between trials |
| **Dopamine Modulators** | Modafinil, DAR-0100A | Modafinil enhanced attention and verbal fluency  DAR-0100A showed moderate improvement in working memory |
| **Acetylcholinesterase Inhibitors** | Galantamine, Donepezil | Showed limited benefits in improving memory |
| **GABAergic Modulators** | Merck L-830982, RL-007 | L-830982 improved working memory, attention, and general cognitive ability  RL-007 reportedly improved general cognitive function and episodic memory (results pending) |
| **Neuropeptides** | Oxytocin | Improved social cognition in several RCTs |
| **Estrogen Receptor Modulators** | Raloxifene | Exhibited pro-cognitive evidence, mainly in working and verbal memory |
| **Others** | Metformin, Valacyclovir, Pentoxifylline, Roflumilast, Cannabidiol, Betahistine | Showed isolated positive results in various cognitive domains |

**Supplemental material- Table 4**

| **Study** | **Intervention** | **Test** |
| --- | --- | --- |
| NCT02832037 | BI 425809 (Iclepertin) | MCCB |
| NCT01047592 | Sarcosine + Benzoate | MCCB |
| NCT03382639 | Luvadaxistat | BACS |
| NCT04822883 | RL-007 | BACS |
| NCT00129441 | Merck L-830982 | RBANS |
| ChiCTR1900021078 | Betahistine | MCCB |
| NCT03271866 | Metformin | MCCB |
| NCT02131129 | rTMS | BACS |
| NCT04055181 | rTMS | RBANS |
| ChiCTR1900024422 | rTMS | MCCB |
| NCT03774927 | rTMS | RBANS |
| UMIN000015953 | tDCS | BACS |
| NCT02128919 | tDCS | MCCB |

**Supplemental material- Fig 1**


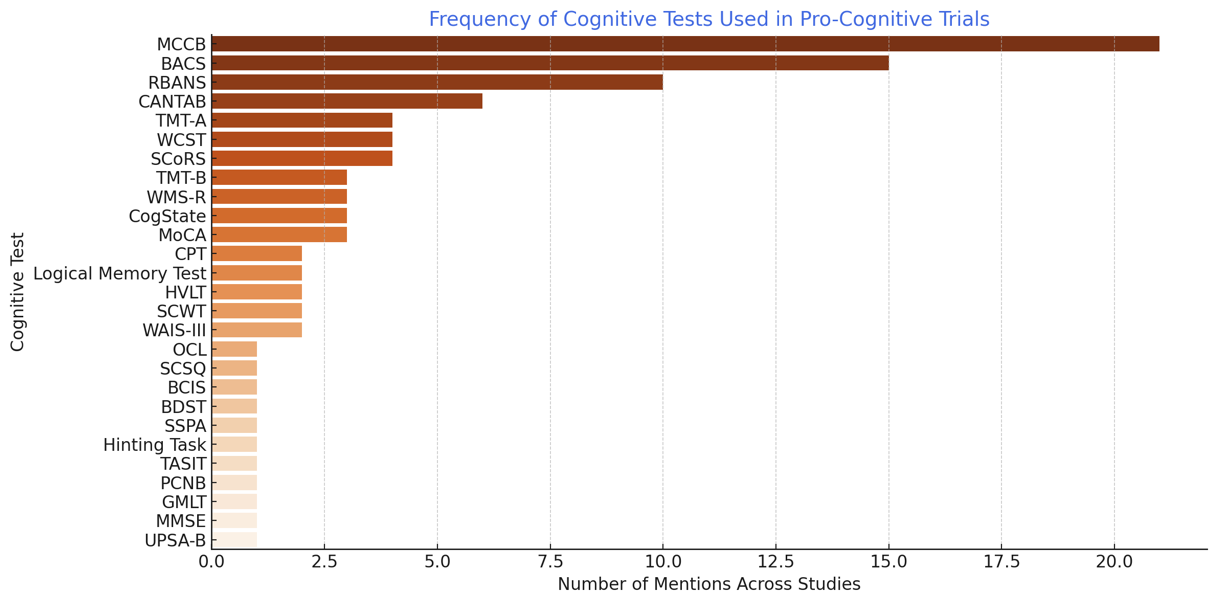


BACS, Brief Assessment of Cognition in Schizophrenia; BCIS, Beck Cognitive Insight Scale; BDST, Backward Digit Span Test; CANTAB, Cambridge Neuropsychological Test Automated Battery; CogState, CogState Cognitive Test Battery; CPT, Continuous Performance Test; GMLT, Groton Maze Learning Test; Hinting Task, Assesses theory of mind (understanding others’ intentions); HVLT, Hopkins Verbal Learning Test; Logical Memory Test, Verbal episodic memory (part of WMS); MCCB, MATRICS Consensus Cognitive Battery; MMSE, Mini-Mental State Examination; MoCA, Montreal Cognitive Assessment; OCL, One Card Learning (CogState); PCNB, Penn Computerized Neurocognitive Battery; RBANS, Repeatable Battery for the Assessment of Neuropsychological Status; SCoRS, Schizophrenia Cognition Rating Scale; SCSQ, Social Cognition Screening Questionnaire; SCWT, Stroop Color and Word Test; SSPA, Social Skills Performance Assessment; TASIT, The Awareness of Social Inference Test; TMT-A, Trail Making Test Part A; TMT-B, Trail Making Test Part B; UPSA-B, UCSD Performance-Based Skills Assessment – Brief; WAIS-III, Wechsler Adult Intelligence Scale – 3rd Edition; WCST, Wisconsin Card Sorting Test; WMS-R, Wechsler Memory Scale – Revised
